# Reassessing the Diagnostic Utility of the Split Hand Index in Amyotrophic Lateral Sclerosis Patients—The Divide by Zero Problem

**DOI:** 10.3390/neurolint14030059

**Published:** 2022-09-01

**Authors:** Florian Antonescu, Ioana Butnariu, Marin Adam, Dana Antonescu-Ghelmez, Sorin Tuță

**Affiliations:** 1 Department of Neurology, “Carol Davila” University of Medicine and Pharmacy, 050471 Bucharest, Romania; 2 Department of Neurology, National Institute of Neurology and Neurovascular Diseases, “Carol Davila” University of Medicine and Pharmacy, 041902 Bucharest, Romania; 3 MedInst Romanian-German Diagnostic Center, 041915 Bucharest, Romania

**Keywords:** amyotrophic lateral sclerosis, split hand index, split hand, severe atrophy

## Abstract

We set out to assess the diagnostic utility of the split hand index (SHI) for amyotrophic lateral sclerosis (ALS) and also to see if and how it can be applied to severely atrophied muscles, a frequent finding in this setting. We enrolled 38 patients from our clinic, 19 diagnosed with ALS and 19 controls, matched for age and sex. The SHI was calculated, on both sides, for all the patients. We calculated a SHI of 0 when the abductor pollicis brevis muscle (ABD) or first dorsal interosseous muscle (FDI) compound muscle action potentials (CMAPs) were unobtainable, and we allotted a value of 0.1 mV to abductor digiti minimi muscle (ADM) CMAP, for mathematical purposes, when the value would have been 0. The means differences were large between groups, with a significant variance heterogeneity. We performed a ROC analysis and obtained an accuracy of 0.83 for a SHI of 7.2, *p*-value < 0.0001. In conclusion, we reaffirm the utility of the SHI in the diagnosis of ALS, especially in limb onset cases, and we think that it can be safely extended to severely atrophied muscles with absent or very low CMAP values, without endangering the sensitivity or specificity.

## 1. Introduction

### 1.1. General Aspects

Amyotrophic lateral sclerosis (ALS) is an idiopathic neurodegenerative disease affecting predominantly the motor neurons that progresses inexorably toward disability and death. Available treatments are only mildly efficacious, with Riluzole holding the spotlight [1]. Although significant progress has been made toward an earlier diagnosis, specific markers are still being sought. A deeper understanding of the complex pathogenesis of ALS is essential in developing earlier diagnostic methods and new treatments [2]. The two seem inextricably linked as the disease is now known to have a significant presymptomatic phase, and any disease modifying intervention has its efficacity limited by the remaining number of viable motor neurons at the start of treatment [3,4].

Electrophysiological studies are indispensable for defining the preclinical stage, but the routine examination is lacking in sensitivity. For example, it is estimated that up to 50% of anterior horn motor neurons may have already been lost until the surface compound muscle action potentials (CMAP) value clearly decreases below the normal [5]. While newer techniques such as motor unit number estimation (MUNE) and motor unit index (MUNIX) show great promise in the research setting [6], they can be quite laborious and could prove difficult to implement in daily medical practice.

### 1.2. The Split Hand Phenomenon

More than 20 years ago, Wilbourn described a phenomenon relatively specific to ALS of asymmetrical atrophy of the muscles of the hand, with preferential wasting of the lateral group and relative sparing of the hypothenar group, which he named “split hand” [7,8].

The typical presentation is that of a slow progressing hand inability and amyotrophies in the thenar eminence and first interosseous space. Signs suggesting diffuse ALS may be absent. The hypothenar eminence is relatively spared. In segmental testing, the index abduction and pollicis abduction are significantly weakened, especially compared with the other small muscles of the hand. The finger flexors are usually spared, including the flexor pollicis longus [9,10]. The patient first complains of losing the pincer grasp, with strong grip movements awkward, but feasible. Later there can be a shift toward an ulnar type of grip in which the patient tends to grasp the objects with the last two fingers against the palm.

Not all ALS patients who present with hand atrophy have this dissociation. In many cases, the atrophies are equal between the lateral and medial hand. This is especially true in bulbar onset cases and advanced disease.

Abductor pollicis brevis (ABD), first dorsal interosseous muscle (FDI), and abductor digiti minimi (ADM) are innervated from the same spinal segments (C8 and T1), but with different nerve pathways, and they also have different cortical motor representations. FDI and ADM are both supplied via the ulnar nerve, APB via the median nerve.

It is unclear why the thenar muscles are preferentially affected in motor neuron disease and the mechanisms may be multifactorial [11]. Several theories have been put forth, all of them centering on the fact that, in humans, the thumb and index finger are used much more intensely than the other fingers. This is thought to lead to greater oxidative stress and metabolic demands for upper and lower motoneurons [12,13].

The corticospinal connections of APB and FDI motor neurons are much more extensive than those of ADM motor neurons, a fact demonstrated by transcranial magnetic stimulation (TMS). This may result in increased glutamate excitotoxicity for the APB and FDI spinal motor neurons [11,13,14]. Using TMS, Menon et al. have demonstrated a global increase in cortical excitability in ALS, but this seems to be unevenly distributed, with significantly higher values for the cortical areas of the APB and FDI muscles [15]. These aspects have led to the interesting hypothesis that the split hand phenomenon may be of cortical origin, but hard evidence remains sparse. Split hand has also been reported in autosomal dominant distal spinal muscular atrophy, spinocerebellar ataxia type 3, and juvenile muscular atrophy, entities that involve only the lower motor neuron, suggesting a peripheral origin [16,17].

Moreover, studies have shown that differential atrophy of the hand occurs with a similar distribution in normal aging, probably the result of greater metabolic demands and differences in intrinsic susceptibility to oxidative stress in the lateral hand region [16]. This finding raises the question of whether the split hand phenomenon in ALS is the result of an accelerated physiological degenerative process.

The different patterns of atrophy affecting the small muscles of the hand can be useful in differentiating ALS from spondylotic amyotrophies. Patients suffering from severe spondylosis develop various deficits and atrophies, but the APB is generally spared [13]. In brachial monomelic amyotrophy (Hirayama disease) a pattern of reversed split hand is often observed, in which ADM is severely affected, and APB spared [18].

### 1.3. The Split Hand Index

There has been significant interest in accurately describing the split hand phenomenon from an electromyographical perspective in a way that will better harness its diagnostic utility. After a few studies that compared the ratios for the CMAP amplitudes of ADM and APB, respectively ADM and FDI, the Split Hand Index (SHI) was described in 2013. It incorporates all three muscles in one index which is calculated by multiplying the CMAP amplitude of the APB with the amplitude of FDI divided by that of the ADM [11,13,19].

In practice, SHI is obtained effortlessly; the examiner has only to add the FDI to the usual nerve conduction studies, which should not prolong the examination by more than a couple of minutes. The active electrode should be positioned to record the maximal CMAP amplitude possible.

The current data support its diagnostic utility, with a 74% sensitivity and 80% specificity in differentiating ALS from other neuromuscular disorders for a SHI value of 5.2. This sensitivity seems to increase without losing in specificity in limb onset patients [19].

MUNIX derived SHI seems to be even better than CMAP SHI [6,20].

The divide-by-zero issue emerged from our daily practice. There are two main uses for the SHI in ALS patients. Firstly, it is an important candidate marker for early diagnosis. We have encountered ALS cases with severe atrophy which was limited initially to one limb, in which the needle examination could not offer sufficient information for advancing the diagnosis beyond possible ALS. The other limb had a normal SHI, and the SHI on the affected limb was impossible to calculate due to the ADM CMAP being 0. The ability to calculate a SHI in such a patient is of great help, especially in differentiating from spondylotic amyotrophies or Hirayama disease.

Secondly, SHI can be used, somewhat to a lesser degree, as a progression marker. ALS cohorts are notoriously difficult to assemble and have a significant dropout rate. Removing longitudinal numerical data as would happen once ADM CMAP reaches 0, would greatly impoverish the possibilities for statistical analysis, as SHI does not stop evolving once ADM CMAP is no longer recordable.

## 2. Materials and Methods

### 2.1. The Study Groups

We enrolled 19 patients diagnosed with ALS according to the Awaji-shima criteria and 19 controls, matched for age and sex, from the neurology clinic of the National Institute of Neurology and Neurovascular Diseases in Bucharest. The control group was selected from patients without any pathology of the central or peripheral motor neurons. All patients provided informed consent, and the study was approved by the “Carol Davila” University of Medicine and Pharmacy scientific research ethics committee.

Each group consisted of 11 women and 8 men, representing approximately 58%, 42%, respectively. Patients were grouped according to the Awaji-shima criteria with 58% having definite ALS, 16% probable and 26% possible ALS. For most patients, the inclusion in the study coincided with the diagnosis of the disease.

### 2.2. Electrophysiological Studies

All examinations were conducted on a Viking EDX 6 system, with EMG EDX software. Upper limb skin temperature was above 31.5 °C in all cases. High and low pass settings were 5 Hz and 10 kHz, respectively. We used 10 mm disc electrodes for motor conduction studies, clip electrodes for sensory nerve conduction, and a 31 mm disc ground electrode.

For motor nerve conduction, we used a belly tendon montage, and the position of G1 was adjusted repeatedly (at least four times) until we obtained a CMAP with maximum amplitude and adequate morphology. We want to stress the importance of actively seeking the correct placement of the active electrode in order to obtain a maximal CMAP. Small deviations in placement can lead to CMAP amplitude variations of up to 20% which completely compromise the technique.

### 2.3. The Split Hand Index

The split hand index was calculated, on both sides for all the patients from the amplitudes of the APB, ADM, and FDI CMAPs, using the formula [19]:

(1) $$SHI=\frac{APB_{CMAP}*FDI_{CMAP}}{ADM_{CMAP}}$$

While other studies did not calculate SHI when any of the CMAP amplitudes was 0, we chose a different approach as is discussed below. We calculated a SHI of 0 when any of the numerator CMAPs were not obtainable, and we allotted a value of 0.1 mV to ADM CMAP for mathematical purposes when the value would have been 0.

### 2.4. Statistical Analysis

For statistical analysis, we used the XLSTAT software [21]. We used the chi-square test of independence and the Pearson coefficient of correlation for the analysis of the level of association for the elements in contingency tables [22]. For analysis of variance, we used the unifactorial ANOVA test. Where the data distribution did not allow for ANOVA, we compared the sets with the nonparametric Mann–Whitney U test. For correlations, we used linear regression analysis, and, in the cases where the data had a nonGaussian distribution, the Spearman correlation test. We used the ROC analysis to establish the predictive accuracy and the cutoff point for various parameters. When we report the *p*-value for the AUC area under the curve, we are referring to the null hypothesis that AUC = 0.5 [22,23]. The means are reported with the standard deviation. We considered a *p*-value < 0.05 statistically significant.

## 3. Results

We calculated the SHI for 18 patients in the study group (in 1 patient we were not able to accurately measure the FDI CMAP) and for all 19 controls.

**Figure 1:**
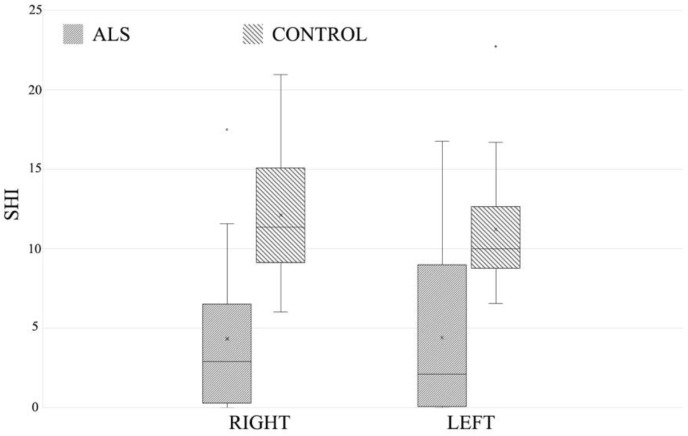
Distribution of the SHI values in the two study groups.

The means for the right and left side, respectively, were very close within each of the two groups: 4.77 ± 5.1 on the right, 4.90 ± 5.04 on the left in ALS patients, respectively, 11.94 ± 3.74 on the right, and 11.19 ± 3.72 on the left in the controls. Comparing the two groups, the differences were large (Figure 1), with significant heterogeneity in the analysis of variance, on the right side F 22.64, *p*-value < 0.01, on the left F 18.78, *p*-value < 0.01.

**Figure 2:**
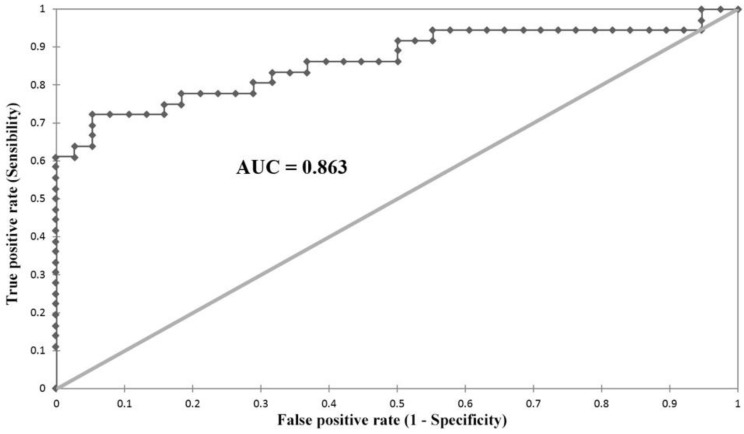
ROC curve evaluating SHI values for the diagnosis of ALS.

We performed a ROC analysis (Figure 2) for evaluating the diagnostic utility and cutoff point for SHI in our study. We obtained a maximum accuracy of 0.83 for an SHI of 7.2 with a sensitivity of 0.72 and a specificity of 0.94, with an AUC of 0.863, with a *p*-value < 0.0001.

We also performed a ROC analysis excluding the divide-by-zero cases. It had the same cutoff point of 7.2, with the same sensitivity of 0.94, a slightly lower specificity of 0.71 (rather than 0.72), the AUC was slightly lower 0.84 (compared to 0.86), the *p*-value was <0.0001 in both cases.

**Figure 3:**
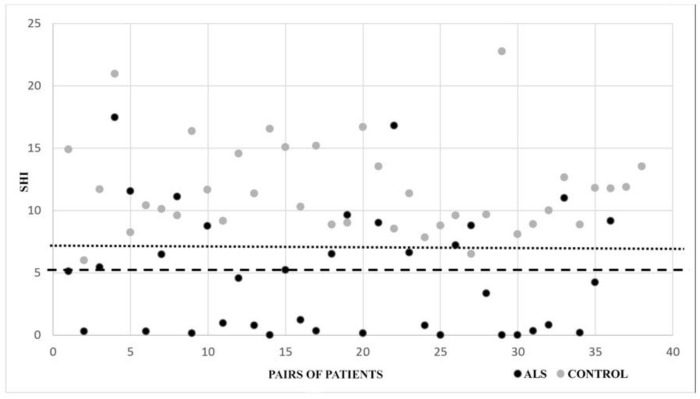
The graphical distribution of all SHI values in the two groups. The dashed line marks the 5.2 cutoff value, and the dotted line the 7.2 cut-off.

The 5.2 cutoff value, shown by Menon et al. in 2013 to have a 74% sensitivity and 80% specificity in distinguishing ALS from other neuromuscular diseases was confirmed by our data [19,24] with a smaller sensitivity (0.58), but a specificity of 1 (58.3% of the SHI recorded in the ALS group were below this point, while none in the control group) as can be seen in (Figure 3) (the dashed and dotted lines mark the cutoffs of 5.2 and 7.2, respectively). This is encouraging as we enrolled patients without neuromuscular disorders in the control group.

In the control group, there was a small tendency of the SHI values to decrease as the patients got older, but it did not bring the values to the pathological territory as defined above.

## 4. Discussion

We consider it useful to calculate the split hand index in ALS suspected patients, as it may offer additional support for the diagnosis, especially in the early stages of the disease. In our study, which compared ALS patients with patients without neuromuscular pathology, the 7.2 value had a sensitivity of 72% and a 94% specificity, with an AUC of 0.863 and high statistical significance. The study by Menon et al. which compared ALS patients with other neuromuscular disorders showed a 5.2 cut-off value with 74% sensitivity and 80% specificity. Thus, it can be argued that SHI values above seven are an argument against the diagnosis of ALS, especially in patients without bulbar involvement.

It remains yet unclear whether the SHI has the same significance in patients with spinal onset versus patients with bulbar onset. In the literature, SHI tends to be lower in spinal onset patients [19,24], while in our patients, the phenomenon was reversed, with values of 4.01 for bulbar onset and 5.13 for spinal onset. Still, the analysis of variance failed to reach statistical significance, probably because of the relatively low number (five) of bulbar onset patients in our cohort.

### 4.1. The Divide-by-Zero Issue

The SHI has a particular divide-by-zero problem when the amplitude of ADM CMAP is 0. The usual approach to arithmetic division by 0 is nonsensical and remains undefined, and the same is true about the fraction 0/0. Thus, equating the lack of a measurable CMAP with an amplitude of 0 mV is problematic in two cases: when the amplitude on ADM is 0 and when the amplitudes on ADM and APB/FDI are both 0. Our view is that EMG is not a mathematical instrument, and its usefulness is greatly increased when the electrophysiological data are analyzed in the correct context.

In our study, the divide-by zero-issue was encountered in 5 of the 18 patients (28%) in the ALS group and in none of the controls. Some 14% of the total number of ADM recordings in ALS patients had a value of 0. Our clinical experience suggests the percentages are probably higher in the clinical setting, as inclusion criteria for ALS research are not met by a significant number of patients. Previous studies chose to exclude patients where there was no response that could be registered on APB or ADM (or the response was smaller than 100 uv) [25]. Excluding the patients from the study is one approach to the problem, but it limits the patient selection for a disease in which enrollment is already difficult and, more importantly, excludes from the analysis the patients with the most severe stages. We think that these patients need not be excluded. Since from a clinical point of view there is no practical difference between patients in which we get no response and the ones in which we obtain small amplitudes of 0.1–0.2 mV, we propose that when the numerator is 0 (CMAP of APB or FDI 0 mV) SHI should have a value of 0. At the same time, where there is no response registered for the ADM CMAP, the denominator should be assigned for mathematical purposes the value of 0.1 mV.

One can argue that substituting the 0 mV value of ADM with 0.1 mV or other small numbers (0.01 or 0.00001 etc.) leads to a situation in which as the ADM CMAP value tends to 0, the SHI increases to aberrant values. Still, the loss of motor units is not a continuous and infinite process but on the contrary discrete and finite. So if we take into consideration the usual motor unit potential (MUP) amplitudes of a few hundred microvolts, we can infer that the minimal CMAP amplitude cannot go to a lower order of magnitude and still be detectable [26,27,28].

We also have to take into consideration that the SHI makes sense only when we are talking about motor neuron disorders (especially ALS). It was developed to quantify a ratio between atrophies of different muscles of the hand which have different speeds of progression and is, practically, an indicator of synchronism. Clinical situations in which a patient has extreme atrophy of one of the ADM, FDI, or APB, with a complete loss of the surface response, while the other two muscles are perfectly preserved are not, in practice, compatible with a C8 anterior horn degeneration. In this case, there are two possibilities: either SHI is 0 (lack of one of the CMAPs on the numerator) or it reaches aberrantly high values from the small value in the denominator (for example values of 6 mV for APB, 10 mV for FDI, 0.1 mV for ADM lead to a SHI of 400). In the first situation, the clinical aspect is not concordant with a neuron motor disease, while in the second the SHI is increased, excluding the possibility of a false-positive diagnosis. In practice, in our study, we have encountered only two situations, the first in which APB or FDI were 0, or the second in which extreme atrophy brought all three values to 0 or very close to it.

If we analyze the case in which APB and FDI have normal amplitudes, and we think of them as constants, SHI becomes a power function dependent on the amplitudes of the ADM CMAP, with the following equation:

(2) $$SHI=c*\;CMAP_{ADM}^{-1}$$

where *c* is a constant.

(3) $$SHI=c*\;CMAP_{ADM}^{-1}$$

**Figure 4:**
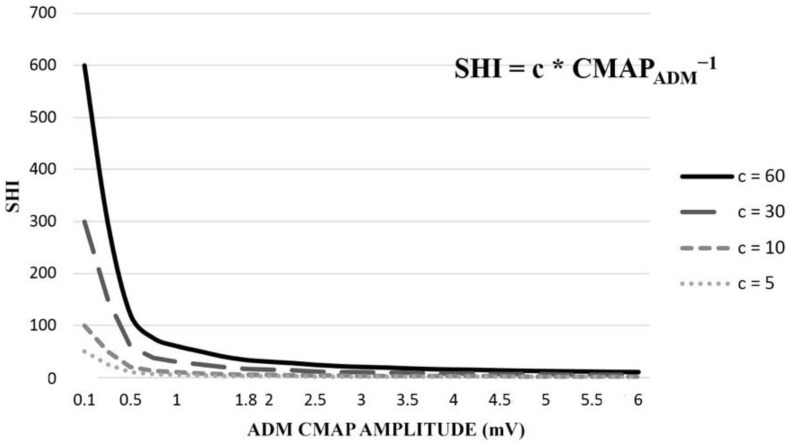
A simulation of SHI values for different ADM CMAP amplitudes. The different lines reflect different values of c (where c is the product of the CMAP amplitudes of APB and FDI).

In Figure 4. we show a simulation for normal values of APB and FDI CMAP of 6 and 10 mV, respectively, varying the ADM CMAP values (the black continuous line). We notice a point of inflection around 0.6–0.8 mV from which small drops in the ADM CMAP amplitude lead to massive increases of SHI. In the same image, we show the curves for SHI for the same ADM CMAP values but for different values of the product of APB and FDI CMAPs (grey dotted lines). As the APB and FDI CMAPs decrease, as is the case in motor neuron disease, we see a slight movement of the inflection point to the left and a linear decrease of the left end of the graph, which practically leaves the range of aberrantly high SHI values.

### 4.2. The MUNIX Experience

Our study also analyzed MUNIX values (manuscript in preparation), and we found them to have good diagnostic accuracy and even to be able to predict needle observed denervation, aspects in concordance with other published research [6,29]. Preliminary data suggest even better accuracy for longitudinal following of disease progression than diagnosis.

Still, despite our favorable results, the experience was marred by difficulties that are surmountable in a research setting but can prove daunting in daily clinical practice. Firstly, while CMAP, SHI, and MUNE refer directly to electrical parameters of the muscle, MUNIX is an index derived from a mathematical model, and in our experience, this makes it more susceptible to examiner bias. The learning curve was significant. Force grading was difficult to standardize between patients and even examiners. This tended to be less significant as the number of analyzed muscles per patient increased and can be addressed by limiting the number of examiners, but the time cost increases notably. Moreover, central motor neuron induced paresis made MUNIX analysis very difficult in some cases and evidently influenced the results in others.

## 5. Conclusions

We consider the SHI a significant addition to the diagnosis of ALS, especially in limb onset cases. Our study shows that it can be safely extended to severely atrophied muscles with absent or very low CMAP values, slightly improving the specificity without endangering the sensitivity. The effect of age on the index seems to be small.

The technique is simple but can be easily flawed if the recording protocol is not strictly respected. We recommend at least four repositionings of the active electrode in order to ensure maximum CMAP amplitude has been recorded for each muscle.

While sensitivity could be higher, we find the specificity adequate. It is our opinion that SHI values below five are a good electrophysiological argument for, and values above seven are a strong argument against the diagnosis of ALS in patients without bulbar involvement.

We are very impressed with and have great hopes for the MUNIX techniques as early diagnostic and progression markers for treatment trials. Still, we think this does not diminish the diagnostic importance of the classic SHI in clinical practice.

## Data Availability

The data presented in this study are available on request from the corresponding author.
